# Autoimmune rhabdomyolysis and a multiorgan display of PD-1 inhibitor induced immune related adverse events during treatment of metastatic melanoma

**DOI:** 10.1186/s40164-019-0140-2

**Published:** 2019-09-10

**Authors:** Hoda Z. Pourhassan, David Tryon, Brett Schaeffer, Hamid Mirshahidi, John Wong

**Affiliations:** 1 Loma Linda University, 11175 Campus Circle, Loma Linda, CA 92354 USA; 2 Loma Linda University, 11234 Anderson St, Loma Linda, CA 92354 USA

**Keywords:** PD-1, Nivolumab, Melanoma, Immunotherapy, Immune mediated adverse events, Myopathy, Rhabdomyolysis

## Abstract

**Background:**

Programmed death-1 (PD-1) inhibitors are among the immunotherapies that have revolutionized our approach to treating several cancers. These novel agents act by blocking PD-1 receptor/PD-1 ligand interactions that would otherwise allow tumor cells to evade host immune destruction by inhibiting response of cytotoxic T-lymphocytes. They are overall well tolerated, though they have been associated with a constellation of immune mediated adverse events (irAEs).

**Case presentation:**

We present a case of rare nivolumab mediated adverse events in a patient with nodular recurrence of melanoma. The patient presented with rhabdomyolysis and shortly thereafter developed a constellation of immune-mediated organ derangements. This case further demonstrates the utility and effectiveness of steroid therapy in the setting of irAEs despite our patient’s eventual poor clinical outcome. While PD-1 inhibitors have revolutionized the treatment of several cancers, they require vigilance by the clinician for early detection and treatment of uncommon but potentially fatal irAEs.

**Conclusions:**

PD-1 inhibitors are now widely used in a multitude of cancer types including melanoma, advanced non-small cell lung cancer, metastatic renal cell carcinoma, and Hodgkin lymphoma amongst others. While these agents are often well tolerated, they are associated with a unique profile of immune-related toxicities that can cause significant morbidity and mortality. Education of both patients and healthcare providers is essential for diagnosis and treatment of these adverse events early in their course. This case highlights the uncommon but potentially serious PD-1-associated toxicity of myopathy and rhabdomyolysis along with other organ involvement and is directly applicable to use of these agents in patients with advanced cancers.

## Introduction

The development of immune checkpoint inhibitors has added a remarkable tool for treatment of patients with advanced cancers. Programmed death-1 (PD-1) inhibitors, like nivolumab, are often described as well tolerated, but have a unique toxicity profile that can be severe and potentially affect multiple organs [1–3]. The most common adverse events involve the skin, GI tract, liver, lungs, and endocrine glands, however, a few cases have been reported involving the heart, eyes, and muscles [1, 2, 4–7]. High-grade musculoskeletal immune-related adverse events (irAEs) are less common and infrequently reported. There is evidence showing that PD-1 inhibitor combination therapy can lead to similar immune-related adverse events as well [8, 9]. Here we report a case of rhabdomyolysis caused by monotherapy with nivolumab in a patient with nodal recurrence of melanoma.

## Case

An 85-year-old man with nodal recurrence of melanoma was admitted to the hospital with generalized weakness and myalgias one week after receiving his second dose of nivolumab. Prior to initiating treatment, patient was described as a very active gentleman with proficient performance status and otherwise good health. He was being treated with 240 mg Nivolumab given day 1 and 15 of 28 day cycles. After completing his first cycle of nivolumab, he began experiencing generalized weakness, myalgia, and fatigue. These symptoms were both persistent and progressive until he became immobile and unable to care for himself leading to emergency room presentation.

On initial evaluation he denied fevers, chest pain, nausea, vomiting, or abdominal pain. He did elicit feeling extremely weak, lightheaded with muscle soreness since his last nivolumab infusion. He was having shortness of breath with exertion and at rest for the past 5 days. He was also having difficulty maintaining balance, experiencing blurry vision, and dry coughs. He had otherwise been active and healthy prior to symptom onset with medical history notable only for hypertension, atrial fibrillation, gastric reflux and coronary artery disease. His labs were most notable for elevated liver enzymes, decreased TSH and elevated free T4. He was also found to have troponin leak, elevated CK-MB and pro BNP concerning for NSTEMI and new onset diastolic heart failure. EKG showed rate-controlled Atrial fibrillation, but no ST changes. Chest x-ray was remarkable for cardiomegaly, pulmonary edema, and a left lung consolidation with effusion. Urine analysis showed large blood.

**Fig. 1:**
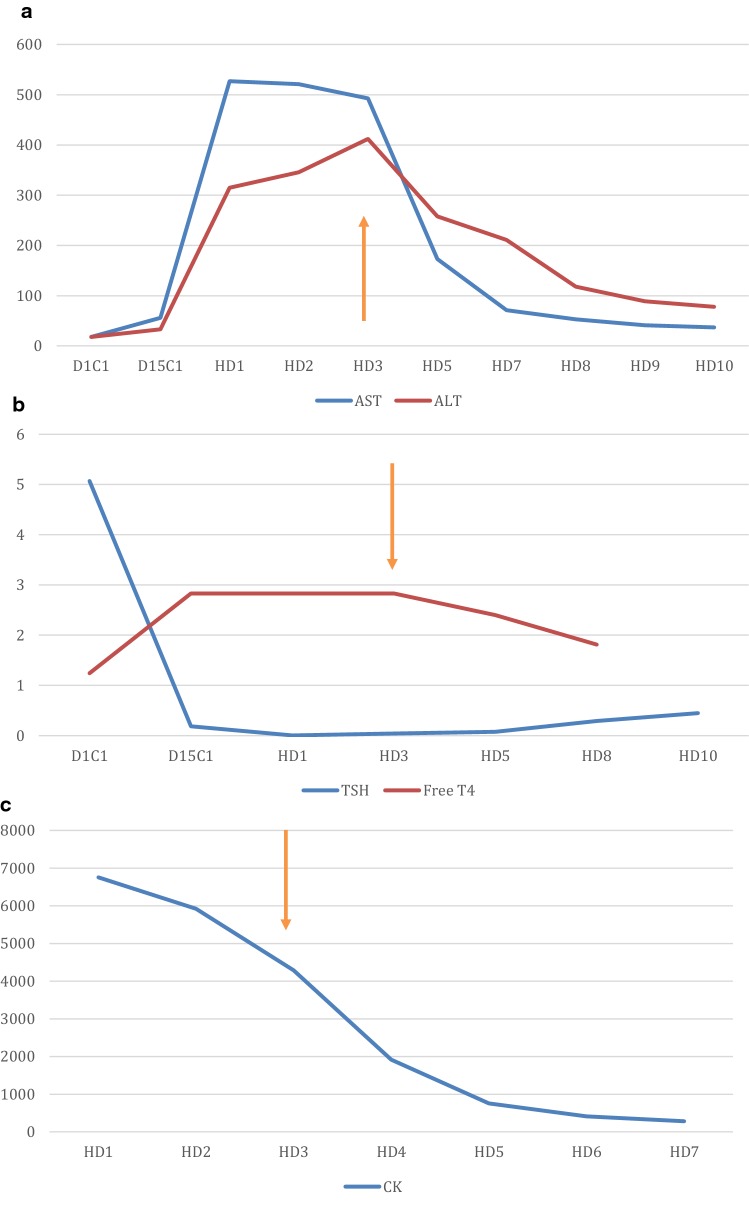
**a** Decline in AST/ALT levels following initiation of steroid therapy (orange arrow). **b** Correction of hyperthyroid state with decline in free T4 and recovery of TSH following initiation of steroid therapy (orange arrow). **c** Decline in CK following initiation of steroid therapy (orange arrow). C1D1, cycle 1 day 1; C2D15, cycle 2 day 15; HD, hospital day

Following admission to cardiology service he was found to have elevated CK and given concern for rhabdomyolysis, he was transferred to the medical intensive care unit and treated with aggressive intravenous fluids. In context of diastolic heart failure and volume overload patient required intubation. Hematology/Oncology was consulted and determined the constellation of symptoms to be irAEs of nivolumab (Table 1). Ophthalmology evaluation including limited eye exam on sedation was notable for keratoconjunctivitis and bilateral abduction deficit. Complete rheumatologic evaluation was negative for other causes of rhabdomyolysis and entire myositis panel was unremarkable. Endocrine workup did not reveal primary endocrine abnormality. Treatment with high dose steroid therapy was started and led to eventual improvement in abnormal laboratory findings (Fig. 1). Despite this, patient continued to deteriorate. He was unable to be weaned from the ventilator and the decision was made to withdraw supportive care.

**Table 1:** Nivolumab and immune related adverse events

irAEs associated with nivolumab	Patient findings
Myalgias and arthralgias	Rhabdomyolysis
Hyperthyroidism	Elevated TSH
Ocular side effects	Keratoconjunctivitis
Hepatotoxicity	Transaminitis
Myocarditis	Troponin leak
Pleural effusion	Left lung consolidation with effusion
Myasthenia Gravis	Progressive weakness (related?)

## Discussion

The development of immune checkpoint inhibitors has changed the management of several types of cancer. Nivolumab and Pembrolizumab, PD-1 monoclonal antibody inhibitors, became FDA-approved for treatment of advanced melanoma in 2014 [10]. As of April 2018, Nivolumab is also approved for treatment of advanced non-small cell lung cancer, metastatic renal cell carcinoma, Hodgkin lymphoma, urothelial carcinoma, metastatic colorectal cancer, hepatocellular carcinoma, and head and neck cancers [10]. And in May 2017, pembrolizumab was approved for the treatment of adult and pediatric patients with unresectable or metastatic, microsatellite instability-high (MSI-H) or mismatch repair deficient tumors. This was the FDA’s first tissue/site-agnostic approval [11].

PD-1 inhibitors act by blocking PD-1 receptor/PD-1 ligand interactions that would otherwise allow tumor cells to evade host immune destruction by inhibiting response of cytotoxic T-lymphocytes [12, 13]. Preventing PD-1 receptor/ligand inhibition enhances host immune response to the tumor. PD-1 inhibitors are often described as well-tolerated, with lower risk of treatment-related adverse events than standard cytotoxic therapy [1, 3]. However, the unique mechanism of action of these immunologic agents can create a distinct toxicity profile caused by excessive T-lymphocyte stimulation. These toxicities are known as immune-related adverse events. The exact pathophysiology for these events is unknown but is presumed to be brought on by a combination of autoreactive T cells, autoantibodies, and/or proinflammatory cytokines such as inrerlekin-17 [14, 15]. The most common side effects involve the skin, gastrointestinal tract, liver, lungs, and endocrine glands, although immune related adverse events could potentially affect any organ [1, 2, 4, 5]. Adverse events are mostly low grade, however high-grade events causing significant morbidity and mortality do occur [1, 2, 4, 5]. Overall, it is not thought that specific tumor types are more or less prone to IAEs as validated through systemic reviews by De Valasco et al. and Khoja et al. However, the latter review did find that tumor histology could perhaps play a role. Patients with melanoma experienced higher incidence of GI and skin irAEs but lower rates of pneumonitis in comparison to NSCLC. Melanoma patients had higher rates of arthritis and myalgias than those with RCC [16, 17]. However, these differences were not adjusted for patient factors such as smoking history and age and could perhaps be additive factors.

In the case presented, musculoskeletal symptoms and rhabdomyolysis were the most prominent findings at presentation. Myalgias and arthralgias are commonly reported adverse events with PD-1 inhibitors, with incidences described as high as 14% [5, 8, 18, 19]. High-grade musculoskeletal side effects are also documented but reporting of musculoskeletal adverse events is inconsistent and incomplete in the literature. Nivolumab-induced myopathy leading to rhabdomyolysis is not commonly reported and is not discussed in many of the landmark studies and meta-analyses on PD-1 inhibitors [3, 5, 8, 19, 20]. The exact frequency of Nivolumab-related myopathy and rhabdomyolysis is not known. Our review of the literature revealed nine reported cases of rhabdomyolysis attributed to nivolumab monotherapy [21–24] and four cases associated with Nivolumab in combination with ipilimumab [6, 7, 9, 25].

Previous cases reporting Nivolumab-related myositis demonstrated myocyte necrosis and extensive infiltration of T-lymphocytes on muscle biopsy [6, 23]. This is consistent with the mechanism of misdirected T-lymphocyte stimulation seen to cause other immune-related adverse events [12, 13]. Cases of myasthenia gravis caused by Nivolumab have also been reported, and several have had concomitant rhabdomyolysis [24, 26–28]. The severe weakness and vision changes seen in this case could fit with an associated myasthenia gravis but testing for acetylcholine receptor antibody was not performed.

Cardiac irAEs are also observed and can be potentially fatal. They can have a variety of manifestations including myocarditis, cardiomyopathy, cardiac fibrosis, heart failure, and cardiac arrest [29, 30]. Analysis of the WHO database revealed 101 individual case safety reports of severe myocarditis following initiation of immune checkpoint inhibitor therapy. 57% of these cases were in patients receiving ant PD-1 monotherapy and in cases with dosing information 64% had received only 1 or two doses of therapy at the time of symptom onset [31]. While neither a significant drop in left ventricular ejection fraction nor chest pain were observed, the elevated troponin and CK-MB seen in this patient may indicate some degree of associated myocarditis [6, 27, 28].

Ocular side effects are uncommon, but include iritis, uveitis, conjunctivitis, keratoconjunctivitis, episcleritis, neuromyolytis optica, and ophthalmoplegia [7, 13, 18, 28]. A full ophthalmologic workup was not able to be performed due to the severity of other illnesses and inability to participate in active exam. However, an initial eye exam demonstrated keratoconjunctivitis and follow up exam was concerning for bilateral abduction defect. Interestingly, a recent case series describing PD-1 inhibitor associated myopathies found that 4 of 8 reported patients with PD-1 inhibitor-associated myopathy and rhabdomyolysis also had ptosis or ophthalmoparesis, and 2 of them had AChR antibodies and were diagnosed with concomitant myasthenia gravis [24]. Despite the uncommon nature of ocular side effects, it is important to recognize that symptoms of eye pain or blurry vision in patients taking PD-1 inhibitors should prompt an ophthalmologic evaluation.

Another suspected immune-related adverse event was the patient’s hyperthyroidism. Endocrine adverse events are seen frequently with immune checkpoint inhibitors, and the rate of thyroid disorders associated with nivolumab is reported to be up to 18% [32, 33]. Hypothyroidism is more prevalent than hyperthyroidism [2, 33]. Prior to this admission, our patient had no documented history of thyroid disease. Testing for thyroid peroxidase antibody was positive, indicating that this patient may have had early Hashimoto’s thyroiditis in a hyperthyroid state. In our case, thyroid function improved rapidly with steroid therapy.

The combination of findings seen in this case as well as the timeline of symptom onset strongly suggest nivolumab-induced irAEs as the cause of symptoms and clinical presentation. This patient developed acute onset of severe weakness, rhabdomyolysis, hyperthyroidism, and blurry vision. He had no medical history of these issues prior to taking nivolumab. No other causes, including rheumatologic or primary endocrine disease related, could be identified for these findings. None of his other active medications had been implicated in rhabdomyolysis. Symptoms started approximately two weeks after the initial cycle of nivolumab and the day after the second cycle. This fits with the finding that most immune-related adverse events occur within 3–6 months of initiation of therapy and can occur within weeks [2]. In addition, the patient’s thyroid dysfunction and rhabdomyolysis responded well to steroid therapy, as would be expected with immune-related adverse events, further solidifying concern for an immune-related etiology.

The treatment of immune-related adverse events associated with PD-1 inhibitors depends on the organ involved and the severity of symptoms. For high-grade adverse events that necessitate reversal of immune-related toxicity, high dose steroids are recommended (prednisone 1–2 mg/kg PO daily or methylprednisolone 1–2 mg/kg IV daily) [18, 34]. Immunotherapy should be discontinued for grade 4 or recurrent grade 3 adverse events [34]. PD-1-associated immune-related adverse events typically respond well to steroids and resolve within 6–12 weeks [34].

These immune related adverse events have recently been defined on a much greater scale. The authors of a September 2018 study retrospectively queried a World Health Organization (WHO) pharmacovigilance database (Vigilyze) comprising more than 16 million adverse drug reactions, and records from seven academic centers [35]. This study found that Anti–PD-1/PD-L1 monotherapy in fact does have a wide distribution of fatal irAEs and underscored the very real risk of complications and death associated with immunotherapies. However, importantly, it also put into perspective the fatality rates of other common oncological interventions such as chemotherapy, stem cell transplantation and other targeted therapies which confer comparable if not greater risk of treatment related fatality.

## Conclusion

PD-1 inhibitors are a remarkable tool for treatment of patients with advanced cancers. While these agents are often well tolerated, they are associated with a unique profile of immune-related toxicities that can cause significant morbidity and mortality. Education of both patients and healthcare providers is essential for diagnosis and treatment of these adverse events early in their course. This case highlights the uncommon but potentially serious PD-1-associated toxicity of myopathy and rhabdomyolysis. It also demonstrates the broad spectrum of organ systems that can be affected by immune therapy. Further studies are needed to determine the prevalence of these events and identify methods to predict and prevent their occurrence.

## Data Availability

Not applicable.

## Abbreviations

PD1: programmed death-1
irAEs: immune mediated adverse events
TSH: thyroid stimulating hormone
T4: thyroxine
CK-MB: creatine kinase-muscle/brain
BNP: brain natriuretic peptide
NSTEMI: non-ST elevated myocardial infarction
EKG: electrocardiogram
CK: creatine kinase
MSI-H: microsatellite instability-high
NSCLC: non-small cell lung cancer
RCC: renal cell carcinoma
WHO: World Health Organization
PO: per os
